# Supplementary material for: Effects of Ketogenic Diet on Quality of Life in Parkinson Disease: An Integrative Review
Source: Nutrients. 2025 Oct 24;17(21):3343. doi: 10.3390/nu17213343 (PMC12609350; doi:10.3390/nu17213343)
Supplement: Supplementary file 1 [file nutrients-17-03343-s001.zip › Supplementary File S1.pdf]

Supplementary File S1

Search Strategy PUBMED

Search Strings	Results
"Ketogenic diet" AND "Parkinson Disease"	32
("Diet, Ketogenic"[Mesh]) AND "Parkinson Disease"[Mesh]	25
(("Diet, Ketogenic"[Mesh]) OR "ketogenic diet") AND ("Parkinson Disease"[Mesh] OR "Parkinson disease")	32
(("Diet, Ketogenic"[Mesh]) OR "ketogenic diet" or “ketosis”) AND ("Parkinson Disease"[Mesh] OR "Parkinson")	104
(("Diet, Ketogenic"[Mesh] OR ("Diet, High-Protein Low-Carbohydrate"[Mesh] OR "Diet, Carbohydrate-Restricted"[Mesh])) AND "Parkinson Disease"[Mesh])	27
(("Diet, Ketogenic"[Mesh]) OR "ketogenic diet") AND ("Parkinson Disease"[Mesh] OR "Parkinson disease") (("Diet, Ketogenic"[Mesh]) OR "ketogenic diet" or "ketosis") AND ("Parkinson Disease"[Mesh] OR "parkinson") AND ("Feasibility Studies"[Mesh])	1
(("Quality of Life"[Mesh]) AND "Diet, Ketogenic"[Mesh]) AND "Parkinson Disease"[Mesh]	0
(("Diet, Ketogenic"[Mesh]) OR "ketogenic diet" or "ketosis") AND ("Parkinson Disease"[Mesh] OR "Parkinson disease") AND "Quality of Life"[Mesh]	2
(("Parkinson Disease"[Mesh]) AND "Diet, Ketogenic"[Mesh]) AND "Signs and Symptoms"[Mesh]	3
(("Diet, Ketogenic"[Mesh]) OR "ketogenic diet" or "ketosis") AND ("Parkinson Disease"[Mesh] OR "Parkinson disease") AND "Signs and Symptoms"[Mesh]	3
(("Diet, Ketogenic"[Mesh]) OR "ketogenic diet" or "ketosis") AND ("Parkinson Disease"[Mesh] OR "Parkinson disease") AND ("Signs and Symptoms"[Mesh] OR "symptoms")	12

Search Strategy COCHRANE

Search Strings	Results
"Ketogenic diet" AND "Parkinson Disease"	20
MeSH descriptor [Diet, Ketogenic] AND MeSH descriptor [Parkinson Disease]	3
(MeSH descriptor [Diet, Ketogenic] OR “ketogenic diet”) AND (MeSH descriptor [Parkinson Disease] OR “Parkinson Disease”)	20
(MeSH descriptor [Diet, Ketogenic] OR MeSH descriptor [Ketosis]) AND (MeSH descriptor [Parkinson Disease] OR “parkinson disease”)	4
(MeSH descriptor [Diet, Ketogenic] OR “ketogenic diet” OR MeSH descriptor [Ketosis]) AND (MeSH descriptor [Parkinson Disease] OR “parkinson disease”)	1

MeSH descriptor [Diet, Ketogenic] OR MeSH descriptor [Diet, High-Protein Low-Carbohydrate] OR MeSH descriptor [Diet, Carbohydrate-Restricted] OR “ketogenic diet”) AND (MeSH descriptor [Parkinson Disease] OR “parkinson disease”)	20
MeSH descriptor [Diet, Ketogenic] AND (MeSH descriptor [Parkinson Disease] OR “parkinson disease”) AND MeSH descriptor [Feasibility Studies]	1
(MeSH descriptor [Diet, Ketogenic] OR “ketogenic diet”) AND (MeSH descriptor [Parkinson Disease] OR “parkinson disease”) AND MeSH descriptor [Quality of life]	0
MeSH descriptor [Diet, Ketogenic] AND MeSH descriptor [Parkinson Disease] AND MeSH descriptor [Symptoms and Signs]	1

Search Strategy CINHAL

**Search Strings**	**Results**
"Ketogenic diet" AND "Parkinson Disease"	26
(MH "Parkinson Disease") AND (MH "Ketogenic diet")	17
(MH "Parkinson Disease" OR "parkinson disease") AND (MH "Diet, Ketogenic" OR "ketogenic diet")	29
(MH "Parkinson Disease" OR "parkinson disease") AND (MH "Diet, Ketogenic" OR "ketogenic diet" OR "ketosis")	32
(MH "Diet, Ketogenic" OR ketogenic diet or keto diet or ketosis or ketogenesis) AND (MH "Parkinson Disease" OR parkinson's disease or parkinson disease or parkinsons disease or pd or parkinsons or parkinsonism)	41
MH "Diet, Ketogenic" AND MH "Quality of Life+" AND MH "Parkinson Disease"	2
MH "Diet, Ketogenic" OR ketogenic diet or keto diet or ketosis or ketogenesis) AND (MH "Parkinson Disease" OR parkinson's disease or parkinson disease or parkinsons disease or pd or parkinsons or parkinsonism) AND (MH "Quality of Life+" OR quality of life or well being or well-being or health-related quality of life)	5
MH "Diet, Ketogenic" AND MH "Quality of Life+" AND MH "Parkinson Disease" AND MH "Symptoms+"	0
((MH "Diet, Ketogenic" OR ketogenic diet or keto diet or ketosis or ketogenesis) AND (MH "Parkinson Disease" OR parkinson's disease or parkinson disease or parkinsons disease or pd or parkinsons or parkinsonism) AND (MH "Quality of Life+" OR quality of life or well being or well-being or health-related quality of life)) AND (MH "Signs and Symptoms+" OR signs and symptoms or clinical manifestations)	0
MH "Diet, Ketogenic" AND MH "Parkinson Disease" AND MH "Symptoms+"	0
((MH "Diet, Ketogenic" OR ketogenic diet or keto diet or ketosis or ketogenesis) AND (MH "Parkinson Disease" OR parkinson's disease or parkinson disease or parkinsons disease or pd or parkinsons or parkinsonism) AND (MH "Signs and Symptoms+" OR signs and symptoms or clinical manifestations)	

MH "Diet, Ketogenic" AND MH "Parkinson Disease" AND feasibility study	0
(((MH "Diet, Ketogenic" OR ketogenic diet or keto diet or ketosis or ketogenesis) AND (MH "Parkinson Disease" OR parkinson's disease or parkinson disease or parkinsons disease or pd or parkinsons or parkinsonism)) AND (feasibility study or feasibility analysis or feasibility assessment)	0

Search Strategy EMBASE

**Search Strings**	**Results**
'ketogenic diet' AND 'parkinson disease'	224
'ketogenic diet' OR ‘low carbohydrate high fat diet’ AND 'parkinson disease'	224
'ketogenic diet' OR ‘low carbohydrate high fat diet’ OR ‘low carbohydrate diet’ AND 'parkinson disease'	231
'ketogenic diet' OR ‘low carbohydrate high fat diet’ OR ‘low carbohydrate diet’ AND 'parkinson disease' AND ‘quality of life’	28
'ketogenic diet' OR ‘low carbohydrate high fat diet’ OR ‘low carbohydrate diet’ AND 'parkinson disease' AND ‘quality of life’ AND “symptomatology”	0
'ketogenic diet' OR ‘low carbohydrate high fat diet’ OR ‘low carbohydrate diet’ AND 'parkinson disease' AND “symptomatology”	4
'ketogenic diet' OR ‘low carbohydrate high fat diet’ OR ‘low carbohydrate diet’ AND 'parkinson disease' AND ‘patient compliance’	2
'ketogenic diet' OR ‘low carbohydrate high fat diet’ OR ‘low carbohydrate diet’ AND 'parkinson disease' AND ‘feasibility study”	6
'ketogenic diet' OR ‘low carbohydrate high fat diet’ OR ‘low carbohydrate diet’ AND 'parkinson disease' AND ‘biological marker’	12
