# Supplementary material for: Effects of Ketogenic Diet on Quality of Life in Parkinson Disease: An Integrative Review
Source: Nutrients. 2025 Oct 24;17(21):3343. doi: 10.3390/nu17213343 (PMC12609350; doi:10.3390/nu17213343)
Supplement: Supplementary file 1 [file nutrients-17-03343-s001.zip › Supplementary File S2.pdf]

Supplementary File S2

JBI Critical appraisal tool for quasi-experimental studies

STUDY	ITEM 1	ITEM 2	ITEM 3	ITEM 4	ITEM 5	ITEM 6	ITEM 7	ITEM 8	ITEM 9	INCLUDE	EXCLUDE	LEVEL (%)	COMMENTS
Tidman et al., 2022	Y	NA	Y	N	Y	Y	Y	Y	Y	X		88%	

Legend: Y=Yes; N=No; U=Unclear; NA=Not Applicable / Items from *JBI Critical appraisal tool for quasi-experimental studies:1=Is it clear in the study what is the 'cause' and what is the 'effect' (i.e. there is no confusion about which variable comes first)?; 2=Were the participants included in any comparisons similar?; 3=Were the participants included in any comparisons receiving similar treatment/care, other than the exposure or intervention of interest?; 4=Was there a control group?; 5=Were there multiple measurements of the outcome both pre and post the intervention/exposure?; 6=Was follow up complete and if not, were differences between groups in terms of their follow up adequately described and analyzed?; 7=Were the outcomes of participants included in any comparisons measured in the same way?;8=Were outcomes measured in a reliable way?; 9=Was appropriate statistical analysis used?*

JBI Critical appraisal tool of analytical cross sectional studies

STUDY	ITEM 1	ITEM 2	ITEM 3	ITEM 4	ITEM 5	ITEM 6	ITEM 7	ITEM 8	INCLUDE	EXCLUDE	LEVEL (%)	COMMENTS
Tidman et al., 2024	Y	Y	Y	Y	U	N	Y	Y	X		75%	

Legend: Y=Yes; N=No; U=Unclear; NA=Not Applicable / Items from Critical appraisal of analytical cross sectional studies: 1= Were the criteria for inclusion in the sample clearly defined? 2=Were the study subjects and the setting described in detail?, 3=Was the exposure measured in a valid and reliable way?, 4=Were objective, standard criteria used for measurement of the condition?, 5=Were confounding factors identified?, 6=Were strategies to deal with confounding factors stated?, 7=Were the outcomes measured in a valid and reliable way?, 8=Was appropriate statistical analysis used?

STUDY	ITEM 1	ITEM 2	ITEM 3	ITEM 4	ITEM 5	ITEM 6	ITEM 7	ITEM 8	ITEM 9	ITEM 10	ITEM 11	ITEM 12	ITEM 13	INCLUDE	EXCLUDE	LEVEL (%)	COMMENTS
Phillips et al., 2018	Y	Y	Y	N	N	Y	Y	Y	U	Y	Y	Y	Y	X		77%	
Krikorian et al., 2019	Y	Y	Y	N	N	Y	Y	Y	U	Y	Y	Y	Y	X		77%	
Koyuncu et al. (2021)	Y	U	Y	N	N	U	Y	Y	U	Y	Y	Y	Y	X		69%	
Choi et al., 2024	Y	Y	Y	Y	Y	Y	Y	Y	U	Y	Y	Y	Y	X		92%	

JBI Critical appraisal tool for Randomized Controlled Trials

Legend: Y=Yes; N=No; U=Unclear; NA=Not Applicable / Items from *JBI Critical appraisal tool for Randomized Controlled Trials: 1.Was true randomization used for assignment of participants to treatment groups?; 2.Was allocation to treatment groups concealed?;3.Were treatment groups similar at the baseline?; 4. Were participants blind to treatment assignment?;5.Were those delivering treatment blind to treatment assignment? ;6.Were outcomes assessors blind to treatment assignment?;7.Were treatment groups treated identically other than the intervention of interest?;8.Was follow up complete and if not, were differences between groups in terms of their follow up adequately described and analyzed?;9.Were participants analyzed in the groups to which they were randomized?;10.Were outcomes measured in the same way for treatment groups?;11.Were outcomes measured in a reliable way?;12.Was appropriate statistical analysis used?;13.Was the trial design appropriate, and any deviations from the standard RCT design (individual randomization, parallel groups) accounted for in the conduct and analysis of the trial?*
